# Four Human Cases of Eastern Equine Encephalitis in Connecticut, USA, during a Larger Regional Outbreak, 2019

**DOI:** 10.3201/eid2708.203730

**Published:** 2021-08

**Authors:** Stacy C. Brown, Justine Cormier, Jessica Tuan, Audun J. Lier, Declan McGuone, Philip M. Armstrong, Firas Kaddouh, Sunil Parikh, Marie Louise Landry, Kevin T. Gobeske

**Affiliations:** Yale University, New Haven, Connecticut, USA (S.C. Brown, J. Cormier, J. Tuan, A.J. Lier, D. McGuone, F. Kaddouh, S. Parikh, M.L. Landry, K.T. Gobeske);; Connecticut Agricultural Experiment Station, New Haven (P.M. Armstrong)

**Keywords:** encephalomyelitis, Eastern equine encephalitis, arbovirus, arbovirus infections, central nervous system viral diseases, disease outbreaks, vector-borne diseases, zoonoses, viruses

## Abstract

During 3 weeks in 2019, 4 human cases of Eastern equine encephalitis (EEE) were diagnosed at a single hospital in Connecticut, USA. The cases coincided with notable shifts in vector–host infection patterns in the northeastern United States and signified a striking change in EEE incidence. All 4 cases were geographically clustered, rapidly progressive, and neurologically devastating. Diagnostic tests conducted by a national commercial reference laboratory revealed initial granulocytic cerebrospinal fluid pleocytosis and false-negative antibody results. EEE virus infection was diagnosed only after patient samples were retested by the arbovirus laboratory of the Centers for Disease Control and Prevention in Fort Collins, Colorado, USA. The crucial diagnostic challenges, clinical findings, and epidemiologic patterns revealed in this outbreak can inform future public health and clinical practice.

## Abstract 2

Incidence increased among human and equine hosts after primary and bridge mosquito virus vectors more than doubled over normal levels 1 month earlier in the season than usual.

Eastern equine encephalitis virus (EEEV) is a single-stranded, positive-sense RNA arbovirus within the *Alphavirus* genus of the *Togaviridae* family. EEEV is maintained in enzootic cycles between ornithophilic *Culiseta melanura* mosquitoes and passerine birds in hardwood swamps in the northeast region of the United States (*1*). Epizootic cycles develop when virus infects mammal-biting bridge vector mosquitoes and then spreads to dead-end hosts, such as humans or husbanded animals (*2*,*3*). Mechanisms of viral or ecologic changes that could sustain epizootic patterns are of great public health interest.

Human Eastern equine encephalitis (EEE) disease develops 4–10 days after arboviral transmission (*4*). Neuroinvasive EEE occurs in just 5% of cases, but mortality rates exceed 30% and neurologic effects are widespread (*5*). Animal studies suggest that central nervous system (CNS) invasion occurs by neuro-olfactory spread or by crossing the blood–brain barrier during peak viremia (*6*). Neuronal injury occurs via direct viral toxicity or secondarily through CNS vasculitis, in which the basal ganglia, thalamus, and cortex commonly are affected (*7*). Histopathologic traits include tissue infiltration of neutrophils and mononuclear cells, perivascular cuffing, inclusion bodies, and neuronal necrosis (*6*–*8*). Like other alphaviruses, EEEV can antagonize components of innate and adaptive immunity to enable rapid propagation in brain tissue (*9*). Inflammatory cascades and direct cytopathy continue to amplify cerebral injury, leading to progressive fever, confusion, coma, cerebral edema, and death (*6*,*10*–*12*).

Thus far, the epidemiologic significance of EEE in the United States has been relatively small, and only 4–8 human cases are diagnosed nationally in a typical year; before 2019, Connecticut had only 1 human case, in 2013 (*13*–*15*). However, during 2019, human cases climbed to 38 nationally, and 19 of these were in New England, representing the largest EEE outbreak in 50 years (*5*,*16*). Experts are closely tracking whether the increased EEE cases reflect similar patterns occurring in West Nile virus (WNV), Powassan virus, Zika virus, and other arboviruses undergoing shifts in background prevalence (*11*–*14*). We describe the diagnosis, clinical features, and epidemiology of 4 human EEE cases from Connecticut, USA, that illustrate lessons for emerging viral disease.

## Cases and Objective Findings

### Case 1

A 77-year-old woman with prior breast cancer and treatment for Lyme disease arrived at the emergency department with acute fever, headache, weakness, and confusion (Appendix Figure 1). Cerebrospinal fluid (CSF) studies revealed mild protein elevation with monocytic pleocytosis (Table). Standard infectious workup was performed, along with WNV testing and an immunofluorescence assay (IFA) arboviral panel that included EEE, Western equine encephalitis, Saint Louis encephalitis, and California encephalitis; all results were negative (Appendix Table). 

**Table:** Clinical and laboratory findings of 4 patients hospitalized with Eastern equine encephalitis, Connecticut, 2019*

Characteristics and diagnostic testing	Case 1			Case 2			Case 3		Case 4		
Age, y/sex	77/F			73/M			64/M		42/M		
Date of illness onset	Aug 28			Sep 11			Sep 12		Aug 21		
Signs and symptoms	Fever, confusion, headache, shock, coma, seizures, flaccid paralysis			Stupor, left-sided weakness			Fever, right arm clumsiness; rapid progression to coma		Neck pain, fever, dysarthria, confusion, seizures		
Day of brain MRI; result	Day 4; diffuse T2 hyperintensity cerebrum, cerebellum, brainstem			Day 4; hyperintensity bilateral basal ganglia, right occipital regions			Day 5; left thalamic enhancement, T2 hyperintensity temporal lobe		Day 3; leptomeningeal enhancement right frontal and parietal lobes, T1 hyperintense signal globi pallidi		
	Days postadmission										
Laboratory findings	3	7	13	2	4	9	2†	4	2	9	21
CSF values											
Protein, mg/dL	90	238	94	112	119	174	108	146	236	288	ND
Glucose, mg/dL	57	74	53	62	64	81	65	78	225	79	ND
Leukocytes/mm^3^	60‡	13	9	428	62	40	1,162	33	343	142	13
Neutrophils, %	22	2	0	86	9	0	76	8	80	0	2
Lymphocytes, %	38	73	100	9	78	85	11	87	14	89	79
Immunoassay, CSF											
Reference lab§											
IgM IFA	–	ND	ND	ND	–	ND	ND	–	ND	–	ND
IgG IFA	–	ND	ND	ND	–	ND	ND	–	ND	–	ND
CDC											
IgM MIA	+	ND	ND	ND	+	ND	ND	+	ND	+	+
PRNT¶	1:4	ND	ND	ND	1:32	ND	ND	1:16	ND	ND	1:4,096#
Immunoassay, serum											
Reference lab**								Day 7			
IgM IFA	–	ND	ND	ND	ND	ND	ND	–	ND	ND	ND
IgG IFA	–	ND	ND	ND	ND	ND	ND	+; 1:16	ND	ND	ND
CDC								Day 6			
IgM MIA	ND	ND	ND	ND	ND	ND	ND	+	ND	ND	ND
PRNT#	ND	ND	ND	ND	ND	ND	ND	ND	ND	ND	ND
Outcome	Death on day 22			Death on day 10			Death on day 8		Severe sequelae		

*CDC, Centers for Disease Control and Prevention; CSF, cerebrospinal fluid; IFA, indirect immunofluorescence assay; lab, laboratory; MIA, microparticle immunoassay; ND, not done; PRNT, plaque reduction neutralization test; –, negative; +, positive. †CSF from outside hospital laboratory before transfer. ‡CSF with 40% mononuclear cells. §CSF IFA >1:4 is positive. ¶CSF PRNT >1:2 is positive. #CSF contaminated with blood. **Serum IFA >1:16 positive.

Despite empiric meningitis treatment, her illness progressed swiftly, involving seizures, coma, flaccid paralysis, and refractory shock, marking an especially severe case (Appendix Figure 1). CSF counts on day 7 and 13 shifted to a lymphocytic pleocytosis with escalating protein level, yet infectious workup results remained negative (Table; Appendix Figure 2). Secondary inflammatory pathology was treated with methylprednisolone, plasma exchange, and intravenous immunoglobulin (IVIg), yielding fleeting small improvements (Appendix Figure 1).

**Figure 1:**
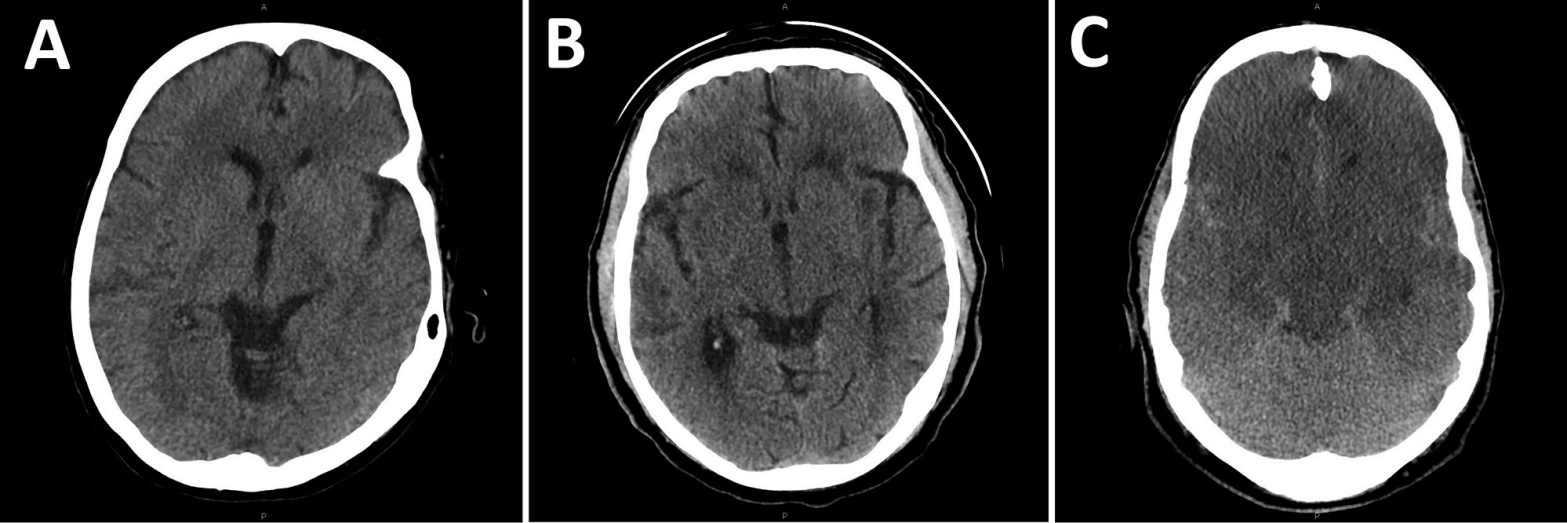
Representative computed tomography axial sections showing early gray-white boundary changes among patients with Eastern equine encephalitis, Connecticut, USA, 2019. A) Axial section showing early gray-white boundary changes on day 3 of illness. B) Axial section with advancing subcortical edema on day 5 of illness. C) Axial section showing diffuse edema with mass effect on adjacent structures and risk of herniation syndromes after 7 days of infection.

**Figure 2:**
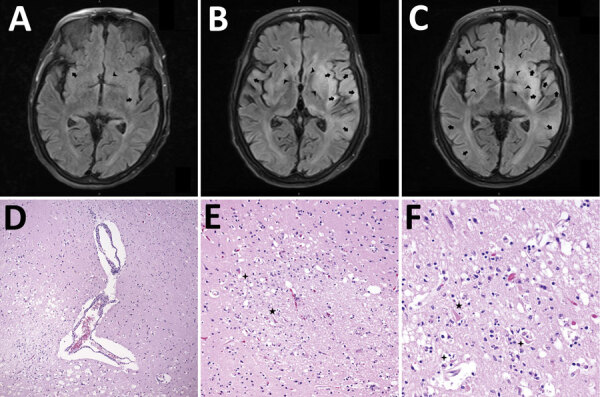
Mechanisms of injury in 4 human cases of Eastern equine encephalitis, Connecticut, USA, 2019. A) Magnetic resonance imaging (MRI) representative axial section from day 2 of a patient’s illness shows early development of edema around the thalamus, basal ganglia, and limbic cortical (arrows) and subcortical (arrowheads) regions. B) Representative MRI axial section from day 4 of a patient’s illness shows progression of injury in these regions and the diencephalon, basal forebrain, and subcortical areas (arrowheads). C) MRI axial section after 1 week of a patient’s illness shows expanding patchy and confluent cortical edema (arrows) and diffuse swelling in basal regions (arrowheads). D) Hematoxylin and eosin (HE)–stained photomicrograph shows the gray-white matter interface with perivascular lymphocytic cuffing and hypoxic-ischemic change in adjacent cortex. Original magnification ×40. E) HE-stained photomicrograph shows a recent gray matter microinfarction, including ischemic neurons with red cell change (5-pointed star) and perineuronal vacuolation (4-pointed star). Original magnification ×200. F) HE-stained photomicrograph shows details of acute hypoxemic-ischemic change with perineuronal (4-pointed stars) and nonspecific vacuolation, red neurons (5-pointed star), rarefaction, and pyknotic cellular debris. Original magnification ×400.

Computed tomography (CT) imaging on day 2 of illness showed nonspecific subcortical changes (Figure 1), but magnetic resonance imaging (MRI) on day 4 showed diffuse T2 signal throughout the forebrain, thalamus, cerebellum, and brainstem (Figure 2). Repeat MRI on days 12 and 18 revealed expansion of bilateral frontotemporal edema and injury, consistent with progression of inflammatory mechanisms (Figure 2; Appendix Figure 2).

Recent mosquito surveillance and reported equine cases of EEE, plus the patient’s temporal and geographic proximity to these reports, environmental exposures, and current symptom progression, all suggested arboviral disease. Thus, we also sent initial CSF samples to the Arbovirus Diagnostic Laboratory, part of the Division of Vector-Borne Diseases, National Center for Emerging and Zoonotic Infectious Diseases, at the Centers for Disease Control and Prevention (CDC) in Fort Collins, Colorado, USA. On day 18 of the patient’s illness, CDC reported microsphere-based immunoassay (MIA) IgM screening for EEEV was positive and confirmed by plaque-reduction neutralization test (PRNT) titers. Given the patient’s grave brain injury, her family elected to pursue comfort-focused care strategies, and she died on day 22 of her illness (Appendix Figure 1).

### Case 2

A 73-year-old man who enjoyed feeding wild animals around his wooded home was found unresponsive after reporting new dizziness, paresthesia, and confusion the previous day. In the emergency department he was stuporous, with left-sided weakness, but neuroimaging results were negative. His CSF samples had a granulocytic pleocytosis and elevated protein on day 2 of illness (Table). Despite empiric treatment, he experienced abnormal neuromuscular tone, brainstem dysfunction, coma, and respiratory failure requiring mechanical ventilation (Appendix Figure 1). Subsequent CSF studies on days 4 and 9 of illness revealed lymphocytic pleocytosis and persistent protein elevation (Table). Commercial EEEV IFA on CSF collected on day 4 was negative, as were further infectious studies. IVIg was started on day 9 without improvement. MRI on day 4 showed diffuse patchy enhancement, edema, and injury (Figure 2).

Retesting of CSF by CDC demonstrated positive EEEV IgM MIA results, confirmed by PRNT on day 9 of illness. Understanding the gravity of his injury, his family requested a comfort-centered care transition, and he died on day 10 after compassionate extubation (Figure 1). At autopsy, gross and microscopic pathology of the brain showed ischemic changes, vascular congestion, inflammatory cell infiltration and microgliosis (Figure 2; Appendix Figure 3), although viral inclusions were not found.

### Case 3

A 64-year-old man with Parkinson’s disease and rheumatoid arthritis was admitted with fever and right arm clumsiness that rapidly progressed to coma and ventilator dependence by day 2. Despite antimicrobial and steroid immunosuppressant treatments, his function declined persistently (Appendix Figure 1). MRI on day 2 and 5 showed progressive enhancement and T2 hyperintensity in limbic, thalamic, and striatal regions, advancing to severe edema and compression (Figure 2).

Initial CSF studies showed elevated protein and granulocytic pleocytosis, shifting to lymphocytic predominance and higher protein by day 4 (Table). New seizures on day 4 were controlled with levetiracetam, but the cooccurring sympathetic and neuromuscular instability remained intractable, signifying especially severe disease (Appendix Figure 1). IVIg was started on day 6 without improvement. On day 8, the patient had acute loss of brainstem reflexes, and a CT showed global cerebral edema and brainstem compression (Figure 1). Hypertonic therapy was started; however, his family soon elected for a comfort-centered focus, and he was compassionately extubated that day.

Reference laboratory EEEV IFA from serum on illness day 6 was negative. Day 4 CSF was retested by CDC; 12 days postmortem, the sample tested positive for EEEV IgM, which was confirmed by PRNT with a 1:16 titer (Table; Appendix Figure 1). Postmortem pathology studies revealed severe ischemic, inflammatory, and compressive injury (Figure 2).

### Case 4

A 42-year-old man with hepatitis C and childhood ventriculoperitoneal shunt placement arrived at the emergency department with neck pain, fever, dysarthria, and confusion. His neurologic function declined rapidly, and he experienced refractory seizures that required intubation and multidrug treatment (Appendix Figure 1). CSF also showed granulocytic to lymphocytic shift of pleocytosis and elevated protein on days 2 and 9 (Table). We confirmed his shunt was nonfunctional; we removed it because of the concern of infection and started the patient on broad-spectrum antimicrobial drugs. Results of autoimmune panels, WNV serology, and EEEV IFA for IgG and IgM from CSF on day 9 were negative (Appendix Table). 

Right frontal lobe brain biopsy on day 15 showed cortical necrosis and inflammation of unclear etiology (Appendix Figure 2). He received empiric IVIg on days 23–27 per regular protocols for neuroinflammatory pathologies but showed no clinical or radiographic improvement. His ongoing hospital course remained complicated (Appendix Figure 1). MRI studies revealed spreading patchy cortical hyperintensity, gyriform enhancement, and mild subcortical injury (Figure 2; Appendix Figure 1, panel C).

On day 40, CDC testing of CSF collected on day 21 of his illness returned positive results for EEEV IgM, confirmed by PRNT titer of 1:4,096. Subsequent CDC retesting of the stored day 9 CSF sample also returned positive results. After 6 weeks, the patient was discharged to inpatient hospice, where he became more alert and regained language comprehension. He moved to a rehab facility and continued to have slow improvement but remained dependent on skilled care.

### Diagnostic Testing

Epidemiologic and clinical features, together with knowledge of unprecedented prevalence of EEEV-positive mosquitoes in the state, prompted our virology laboratory to contact CDC’s Arbovirus Diagnostic Laboratory to have case 1 retested with expedited processing. After this positive test result, the Connecticut Department of Public Health (DPH) and CDC approved submission of subsequent samples directly to CDC for priority testing. Turnaround was improved from 3–4 weeks to <10 days. Connecticut DPH subsequently validated an in-house EEEV IgM MIA test for rapid and sensitive screening for future outbreaks.

Reference laboratory EEEV IFA testing of CSF at 1:4 dilution was negative for IgM for all cases in samples spanning day 3, 4, 6, and 9 of illness. Upon learning of false-negative results, the commercial laboratory retested 3 of the CSF samples undiluted, but results remained negative. Thus, for our samples, IFA IgM findings did not correlate with the duration of illness or concentration as shown by PRNT titer.

### Local Epidemiology

**Figure 3:**
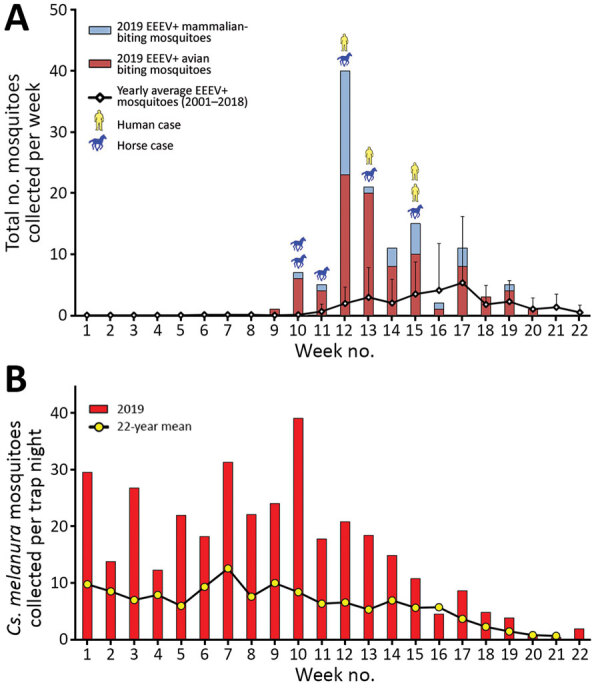
Epidemiology of EEE and mosquito vector populations, Connecticut, USA, June 2–November 2, 2019. A) Epidemic curve of EEE in Connecticut in mosquito populations, horses, and humans. Error bars indicate 95% CIs. B) Weekly collection of *Culiseta melanura* mosquitoes during 2019 compared with long-term historical averages. EEE, Eastern equine encephalitis; EEEV, EEE virus; +, positive.

**Figure 4:**
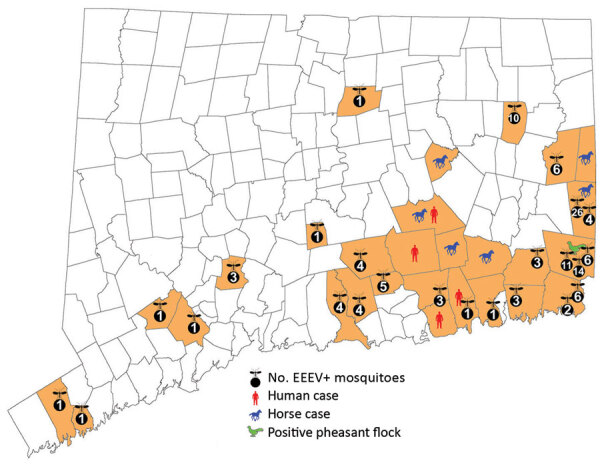
Geographic distribution of EEE in mosquitoes, humans, horses, and pheasant flocks, Connecticut, USA, 2019. EEE, Eastern equine encephalitis; EEEV, EEE virus; +, positive.

During June–October 2019, mosquitoes were trapped and tested for arbovirus infection at 92 fixed-trapping sites in Connecticut as a part of the statewide surveillance program. During the season, mosquitoes are collected weekly at each location by using CDC light traps baited with dry ice and gravid traps baited with a hay-lactalbumin infusion. Sample preparation and EEEV detection was consistent for all sites and between years. Connecticut surveillance sites first collected EEEV-positive mosquitoes in late July 2019, ≈4 weeks earlier than other years having EEEV (*17*,*18*). Equine EEEV infections surfaced in early August and continued through September. Numbers of EEEV-positive mosquitoes peaked by late August, but numbers remained elevated through mid-October. The 4 human cases occurred in late August and early September within a localized region of southeastern Connecticut where equine and vector involvement also were highest. Shortly before the incidence in humans, numbers of *Cs. melanura* mosquitoes and mammal-biting bridge vectors carrying EEEV both rose distinctly (Figure 3, panel A). Climate conditions in the preceding months had shown temperatures 2.4°F above average through the summer and 2.6°F warmer during the winter; the region had 11 inches more precipitation than normal (*19*,*20*). Concordantly, 21,880 *Cs. melanura* mosquitoes were collected in Connecticut during 2019, which is 2.4 times the annual average during 2001–2018 (Figure 3, panel B). All human and equine EEE cases were tightly clustered geographically and coincided with temperature and vector population rises (Figure 4).

## Discussion

The cases we report represent a notable diversion from the background incidence and clinical severity of EEE in this region. This single-state experience is striking individually but becomes more salient in relation to patterns occurring contemporaneously in nearby states and possibly in the future (Appendix Figure 5). Recognizing and controlling epidemics requires dependable diagnostic methods and coordination between clinicians, health departments, and surveillance programs. Viral neuroinvasive infections can pose even greater challenges because our best diagnostic efforts reveal an etiology in only one third of encephalitis cases (*21*). Our experience demonstrates the importance of unified efforts in recognizing a new epidemic and avoiding public health pitfalls.

Because virus rarely is present in specimens when patients are symptomatic, EEEV assays target host antibodies produced against viral epitopes. EEEV IgM usually becomes measurable 3–8 days after infection (*22*). Assay processing time can further extend the lag time from clinical onset to initiation of secondary injury mechanisms and the ability to make diagnostically informed decisions. The cases we report demonstrate the challenges of mismatched timeframes for analytic pathways versus critical periods for intervention in patient care and community education. Assessment of data needed for decision-making becomes crucial, especially when considerations involve rapidly devastating illness, expensive treatments, or time-sensitive community interventions. Our experience exemplified the need for prompt compilation and synthesis of findings to guide decisions, such as whether and when to begin IVIg or plasma exchange treatments during case 1 and to postpone outdoor school sporting events statewide during case 4. Future ability to establish evidence-based treatments or targeted protocols likewise will depend on improved timing of diagnosis and decision-making.

Shared clinical features from our patients also highlight patterns to alert practitioners of EEEV infection as the etiology of encephalitis. We noted rapid shifts in CSF profiles from granulocytic to lymphocytic predominance. Seizures, secondary inflammatory injury, cerebral edema, rapid deterioration, and clinical considerations for starting immunomodulatory treatments all should be signals prompting outreach to public health and laboratory medicine colleagues. Other features of severe disease that were especially prominent in our patients, and possibly underrecognized as elements of critical neuroinvasive EEE overall, included refractory shock with adrenergic insensitivity and neuromuscular instability with either flaccid paralysis or rigidity.

Diagnosing EEE in our patients was unexpectedly challenging because the commercially available arbovirus IFA test failed to detect EEEV antibodies in all cases. Yet, all 4 CSF samples tested positive at the CDC laboratory by the more recently developed MIA to screen for EEEV IgM and confirmatory PRNT. Whereas older IFA methods use spots of virus-infected cells affixed to slide wells, MIA uses microbeads coated with EEEV envelope proteins as the antigen-presenting substrate incubated with a patient’s CSF or serum, then secondary IgM for detection (*23*). For PRNT, IgM-reactive samples are serially diluted, each dilution is mixed with infectious virus, then inoculated into cell culture. If present, virus-specific neutralizing antibodies will reduce the number of virus-induced plaques observed after a designated incubation period. Of note, the virus strain EEE New Jersey 60 is used in all 3 methods and does not appear to explain the discrepancy.

Because only 1 test in the United States, DxSelect Arbovirus IFA IgM/IgG (Focus Diagnostics, https://www.focusdx.com), has been cleared by the Food and Drug Administration for diagnosing EEEV, Western equine encephalitis virus, St. Louis encephalitis virus, and California encephalitis/La Crosse virus, all commercial reference laboratories use the same IFA kits for arbovirus antibody detection. Many potential variables exist within IFA testing, including slide and reagent manufacturing, manual processing of antibody application steps, microscopy techniques and equipment, and subjective reading of results. Nevertheless, no other false-negative IFA results have been reported to date. Of note, arboviral IFA kits are approved by the Food and Drug Administration only for serum testing at an initial dilution of 1:16; CSF testing must be validated independently at each commercial laboratory, including ascertaining the starting dilution. Because of the failure to detect antibody at a 1:4 screening dilution in our 4 cases, the reference laboratory now screens all CSF samples undiluted.

Because only 4–8 cases occur nationally in a typical year, EEE is a rarely diagnosed infection, and large reference laboratories might receive few to no positive samples annually. The infrequent positivity rates among samples provides little opportunity to verify diagnostic assays by using clinical specimens or for comparison between IFA and other methods; we found no such reports in the literature. Nonetheless, MIA clearly was more sensitive than IFA as a screening test in our patients. A specific reason for the failure of IFA testing in these cases was not identified, but the presumed lower sensitivity of IFA methods should remain a consideration in future epidemics. Regardless of the cause, the discovery of systematic false-negative results highlights the need to think broadly about testing strategies for arboviral disease in a public health context, and particularly for cases of infectious encephalitis.

Crucial epidemiologic and viral ecologic factors also shed light on regional EEE emergence and could provide warnings for EEE risk in future years. Historically, EEEV has cyclic years of high amplification; Connecticut saw spikes in 2003 and 2009 and in 2013, when the only prior human case was recorded (*15*,*18*). However, closer examination of mosquito surveillance during high-activity years reveals patterns associated with the emergence of epidemics (Figure 3; Appendix Figure 4). High-activity years had exceptional increases of EEEV carriage by *Culiseta* mosquitoes, after which greater infection of mammal-biting vectors was reported. When human or equine cases emerged, temporal and geographic correlation were noted after the upsurges (Figures 3, 4). Mechanisms for human spillover from vector–host cycles remain unclear; studies also show direct transmission from primary enzootic vectors to mammalian and human hosts during larger EEE epizootic events (*1*–*3*,*24*–*27*). Therefore, prevention must be informed by recognition of earlier seasonal escalation of *Cs. melanura* populations and rapid rise of EEEV within enzootic or epizootic vectors (*24*).

Locally, the Connecticut River Valley has abundant densely wooded freshwater swamps, creating ideal habitat for EEEV enzootic vectors and hosts, and likely models conditions elsewhere (*1*,*17*). Weather patterns preceding our cases increased the productivity of mosquito larval environments and might have fostered *Cs. melanura* mosquitoes overwintering and early reproduction, per our trap collection timelines (*1*,*2*,*18*–*20*,*27*). Indeed, EEEV-positive mosquito counts were greater than any other arbovirus in our region, reaching 20 times normal in Connecticut and 200 times normal in Massachusetts (Appendix Figure 4). As the climate warms, the risk for EEE outbreaks could increase because of emergence of EEEV into optimized environments and from late-season persistence of infected vectors. Additional studies assessing population genetics of the virus and vectors are needed to illuminate the triggers and evolution of such epidemics (*2*,*5*,*25*–*27*).

In the face of climatic and global changes, including warmer temperatures and human population growth and interaction with vector ecologies, future arboviral epidemics are certain, and the likelihood of an increasing burden of EEE is high. Coordination between public health and hospital settings to improve surveillance, clinical detection, and community education will be essential for gaining control of this potentially devastating neuroinvasive disease. Of note, awareness to reappraise and navigate diagnostic testing through local and reference laboratories has become a crucial skill for early detection of EEE cases and management of a local epidemic. Our state’s experience shows the importance of bringing together public health, healthcare, diagnostic systems, and vector-control agencies, as well as community education and diagnostic systems, to mitigate risk for EEE among the public.

Appendix Additional information on 4 human cases of Eastern equine encephalitis, Connecticut, USA, 2019.

## References

[1] Molaei G, Thomas MC, Muller T, Medlock J, Shepard JJ, Armstrong PM, et al. Dynamics of vector-host Interactions in avian communities in four Eastern equine encephalitis virus foci in the Northeastern U.S. PLoS Negl Trop Dis. 2016;10:e0004347. 10.1371/journal.pntd.0004347 26751704 PMC4713425

[2] Arrigo NC, Adams AP, Weaver SC. Evolutionary patterns of eastern equine encephalitis virus in North versus South America suggest ecological differences and taxonomic revision. J Virol. 2010;84:1014–25. 10.1128/JVI.01586-09 19889755 PMC2798374

[3] Heberlein-Larson LA, Tan Y, Stark LM, Cannons AC, Shilts MH, Unnasch TR, et al. Complex epidemiological dynamics of Eastern equine encephalitis virus in Florida. Am J Trop Med Hyg. 2019;100:1266–74. 10.4269/ajtmh.18-0783 30860014 PMC6493969

[4] Gill CM, Beckham JD, Piquet AL, Tyler KL, Pastula DM. Five emerging neuroinvasive arboviral diseases: Cache Valley, Eastern equine encephalitis, Jamestown Canyon, Powassan, and Usutu. Semin Neurol. 2019;39:419–27. 10.1055/s-0039-1687839 31533182

[5] Morens DM, Folkers GK, Fauci AS. Eastern equine encephalitis virus—another emergent arbovirus in the United States. N Engl J Med. 2019;381:1989–92. 10.1056/NEJMp1914328 31747726

[6] Vogel P, Kell WM, Fritz DL, Parker MD, Schoepp RJ. Early events in the pathogenesis of eastern equine encephalitis virus in mice. Am J Pathol. 2005;166:159–71. 10.1016/S0002-9440(10)62241-9 15632009 PMC1602312

[7] Calisher CH. Medically important arboviruses of the United States and Canada. Clin Microbiol Rev. 1994;7:89–116. 10.1128/CMR.7.1.89 8118792 PMC358307

[8] Roy CJ, Reed DS, Wilhelmsen CL, Hartings J, Norris S, Steele KE. Pathogenesis of aerosolized Eastern Equine Encephalitis virus infection in guinea pigs. Virol J. 2009;6:170. 10.1186/1743-422X-6-170 19852817 PMC2770496

[9] Trobaugh DW, Klimstra WB. Alphaviruses suppress host immunity by preventing myeloid cell replication and antagonizing innate immune responses. Curr Opin Virol. 2017;23:30–4. 10.1016/j.coviro.2017.02.004 28288385 PMC5823529

[10] Deresiewicz RL, Thaler SJ, Hsu L, Zamani AA. Clinical and neuroradiographic manifestations of eastern equine encephalitis. N Engl J Med. 1997;336:1867–74. 10.1056/NEJM199706263362604 9197215

[11] Hollidge BS, González-Scarano F, Soldan SS. Arboviral encephalitides: transmission, emergence, and pathogenesis. J Neuroimmune Pharmacol. 2010;5:428–42. 10.1007/s11481-010-9234-7 20652430 PMC3286874

[12] Zacks MA, Paessler S. Encephalitic alphaviruses. Vet Microbiol. 2010;140:281–6. 10.1016/j.vetmic.2009.08.023 19775836 PMC2814892

[13] Lindsey NP, Staples JE, Fischer M. Eastern equine encephalitis virus in the United States, 2003–2016. Am J Trop Med Hyg. 2018;98:1472–7. 10.4269/ajtmh.17-0927 29557336 PMC5953388

[14] McDonald E, Martin SW, Landry K, Gould CV, Lehman J, Fischer M, et al. West Nile virus and other domestic nationally notifiable arboviral diseases—United States, 2018. MMWR Morb Mortal Wkly Rep. 2019;68:673–8. 10.15585/mmwr.mm6831a1 31393865 PMC6687196

[15] Nelson R, Ciesielski T, Andreadis T, Armstrong PM. Human case of Eastern equine encephalitis–Connecticut, 2013. Connecticut Epidemiologst. 2014;34:9–10 [cited 2020 Aug 8]. https://portal.ct.gov/-/media/Departments-and-Agencies/DPH/dph/infectious_diseases/CTEPINEWS/Vol34No3pdf.pdf

[16] Lindsey NP, Martin SW, Staples JE, Fischer M. Notes from the field: multistate outbreak of Eastern equine encephalitis virus—United States, 2019. MMWR Morb Mortal Wkly Rep. 2020;69:50–1. 10.15585/mmwr.mm6902a4 31945032 PMC6973353

[17] Skaff NK, Armstrong PM, Andreadis TG, Cheruvelil KS. Wetland characteristics linked to broad-scale patterns in *Culiseta melanura* abundance and eastern equine encephalitis virus infection. Parasit Vectors. 2017;10:501. 10.1186/s13071-017-2482-0 29047412 PMC5648514

[18] The Connecticut Agricultural Experiment Station. State of Connecticut mosquito trapping and arbovirus testing program. 2019 Oct 22 [cited 2020 March 28]. https://portal.ct.gov/CAES/Mosquito-Testing/Introductory/State-of-Connecticut-Mosquito-Trapping-and-Arbovirus-Testing-Program

[19] Mermel LA. Association of human Eastern equine encephalitis with precipitation levels in Massachusetts. JAMA Netw Open. 2020;3:e1920261. 10.1001/jamanetworkopen.2019.20261 32003815 PMC7042854

[20] National Oceanic and Atmospheric Administration. 1981–2010 U.S. climate normals. 2018 Nov 5 [cited 2020 Mar 8]. https://www.ncdc.noaa.gov/data-access/land-based-station-data/land-based-datasets/climate-normals/1981-2010-normals-data

[21] Bloch KC, Glaser CA. Encephalitis surveillance through the emerging infections program, 1997–2010. Emerg Infect Dis. 2015;21:1562–7. 10.3201/eid2109.150295 26295485 PMC4550161

[22] Centers for Disease Control and Prevention. Arboviral diseases, neuroinvasive and non-neuroinvasive case definition 2015; updated 2017 Aug 2 [cited 2020 March 20]. https://wwwn.cdc.gov/nndss/conditions/eastern-equine-encephalitis-virus-disease/case-definition/2015

[23] Johnson AJ, Noga AJ, Kosoy O, Lanciotti RS, Johnson AA, Biggerstaff BJ. Duplex microsphere-based immunoassay for detection of anti-West Nile virus and anti-St. Louis encephalitis virus immunoglobulin m antibodies. Clin Diagn Lab Immunol. 2005;12:566–74. 15879016 10.1128/CDLI.12.5.566-574.2005 PMC1112082

[24] Molaei G, Armstrong PM, Graham AC, Kramer LD, Andreadis TG. Insights into the recent emergence and expansion of eastern equine encephalitis virus in a new focus in the Northern New England USA. Parasit Vectors. 2015;8:516. 10.1186/s13071-015-1145-2 26453283 PMC4600208

[25] Soghigian J, Andreadis TG, Molaei G. Population genomics of *Culiseta melanura*, the principal vector of Eastern equine encephalitis virus in the United States. PLoS Negl Trop Dis. 2018;12:e0006698. 10.1371/journal.pntd.0006698 30118494 PMC6114928

[26] Shepard JJ, Andreadis TG, Thomas MC, Molaei G. Host associations of mosquitoes at eastern equine encephalitis virus foci in Connecticut, USA. Parasit Vectors. 2016;9:474. 10.1186/s13071-016-1765-1 27577939 PMC5006286

[27] Tan Y, Lam TT, Heberlein-Larson LA, Smole SC, Auguste AJ, Hennigan S, et al. Large-scale complete-genome sequencing and phylodynamic analysis of Eastern equine encephalitis virus reveals source-sink transmission dynamics in the United States. J Virol. 2018;92:e00074–18. 10.1128/JVI.00074-18 29618651 PMC5974483

