# Supplementary material for: Four Human Cases of Eastern Equine Encephalitis in Connecticut, USA, during a Larger Regional Outbreak, 2019
Source: Emerg Infect Dis. 2021 Aug;27(8):2042–51. doi: 10.3201/eid2708.203730 (PMC8314835; doi:10.3201/eid2708.203730)
Supplement: Appendix — Additional information on 4 human cases of Eastern equine encephalitis, Connecticut, USA, 2019. [file 20-3730-Techapp-s1.pdf]

Article DOI: https://doi.org/10.3201/eid2708.203730

Four Human Cases of Eastern Equine Encephalitis in Connecticut, USA, during a Larger Regional Outbreak, 2019

Appendix

Appendix Table. Laboratory diagnostic evaluations by date for 4 patients in whom Eastern equine encephalitis eventually was diagnosed, Connecticut, USA, 2019*

Patient 1	Patient 2	Patient 3	Patient 4
Hospital day 1<br>Blood tests: culture ×2 (–); *Babesia* smear (–); Lyme antibody (–); *Anaplasma* PCR (–)	Hospital day 1<br>Blood tests: cultures (–) ×2; *Anaplasma phagocytophilium* PCR (–); *Babesia* smear (–)<br><br>Urine culture (–)	Hospital day 1<br>Blood tests: cultures ×2 (–)	Hospital day 1<br>Blood tests: *Babesia* smear (–); Lyme antibodies (–); PCR for *Ehrlichia* and *Anaplasma* species (–)
Hospital day 2<br>Serum tests at an outside reference laboratory: EEEV IgG <1:16, IgM <1:16 (–)<br><br>CSF test at an outside reference laboratory: EEEV IgG <1:4 (–), IgM <1:4 (–)<br><br>Blood tests: *Toxoplasma* IgM and IgG; CMV (–); *Cryptococcal* antigen (–); HIV-1/HIV-2 4th generation antibody/antigen screen (–); EBV VCA IgG (+), VCA IgG (–), and EBNA-1 IgG (–); bacterial culture (–)<br><br>CSF at Mayo Clinic: AFB culture (–); encephalopathy autoimmune evaluation (–)<br><br>CSF tests: flow cytometry (–); *Listeria* antibody (–); adenosine deaminase(–); encephalopathy autoimmune evaluation (–);and PCR for EBV (–); CMV (–); Powassan virus (–); HSV (–); and VZV (–)	Hospital day 1<br>Blood tests: *Leptospira* IgM (–); *Leptospira* DNA, qualitative (–)<br><br>CSF IFA at an outside reference laboratory: EEEV IgG <1:4 (–), IgM <1:4 (–)<br><br>CSF tests: bacterial culture (–); rabies titer (–); WNV antibody IgG and IgM (–); and PCR for VZV (–) and HSV (–)<br><br>Respiratory samples: virus PCR panel (–); lower respiratory culture, normal flora	Hospital day 2<br>Blood tests: TB† (–)	Hospital day 2<br>CSF test: bacterial culture (–)
Hospital day 3<br>Blood test: LCMV IgM and IgG (–)<br><br>Lower respiratory culture (–)	Hospital day 3<br>Blood tests: HIV-1/HIV-2 4th generation antibody/antigen screen (–)	Hospital day 3<br>CSF tests: bacterial culture (–); AFB culture (–); WNV IgG and IgM (–); *Listeria* antibody (–); Powassan IgM (–); and PCR for CMV (–); adenovirus (–); HSV (–); enterovirus (–); VZV (–); JCV (–); and HHV-6 (–)<br><br>Sputum culture: normal flora<br><br>CSF IFA at outside reference laboratory: EEEV IgG <1:4 (–), IgM <1:4 (–)	Hospital day 3<br>CSF tests: PCR for HSV (–); VZV (–); and CMV (–)

Patient 1	Patient 2	Patient 3	Patient 4
Hospital day 4<br>Blood test: WNV IgM and IgG blood (–)<br><br>Stool test: *C. difficile* assay (–)	Hospital day 4<br>Lower respiratory culture, normal flora<br><br>Blood tests: TB† (–); cultures ×2 (–); *Ehrlichia* and *Anaplasma* species PCR (–); *Anaplasma phagocytophilum* PCR (–); *Bartonella Henselae* Ab IgM and IgG (–); Lyme Ab (+) 1.55 LI, IgM and IgG WBs (–); metagenomics at UCSF (–)<br><br>CSF tests: bacterial culture (–);fungal culture (–); cytology hypercellular with abundant lymphocytes; flow cytometry with monoclonal B cell lymphoproliferative disease, 6%–7% cellularity; *Listeria* antibody (–); *Toxoplasma gondii* PCR (–); adenosine deaminase (–; 3.2 U/L); AFB culture (–); *Cryptococcal* antigen (–); *Tropheryma whipplei* DNA PCR (–)<br><br>CSF tests at outside laboratory: arbovirus antibody panel IgM and IgG (–) at an outside reference laboratory	Hospital day 4<br>Blood tests: Lyme antibody blood (–); *Toxoplasma gondii* IgG (–)<br><br>Sputum culture: normal flora	Hospital day 4<br>Sputum culture: normal flora
Hospital day 5<br>Blood test: *Treponema pallidum* antibody (–)	Hospital day 5<br>Blood tests: *Treponema pallidum* Ab (–); ANCA (–); ANA (–)	Hospital day 5<br>Stool test: *C. difficile* assay stool (–)<br><br>Serum test at outside reference laboratory: EEEV IgG <1:16 (–), IgM <1:16 (–)	Hospital day 6<br>Blood tests: *Clostridium difficile* assay (–); culture (–)<br><br>Sputum culture: normal flora
Hospital day 6<br>Lower respiratory culture: *Aspergillus* species (not fumigatus/flavus)<br><br>Blood culture (–)<br><br>CSF bacterial culture (–)<br><br>CSF PCR: WNV (–); Enterovirus (–); HSV (–); HHV-6 (–)	Hospital day 6<br>Blood tests: vitamin B1 level, low <6 nmol/L; *Cryoglobulin* (–)	Hospital day 7<br>Tissue tests: PCR for VZV (–); and HSV (–)<br><br>Respiratory viral panel (–)<br><br>Deep wound culture 1 CFU *Pseudomonas aeruginosa*<br><br>Autopsy of the brain	Hospital day 7<br>Blood tests: HIV-1/HIV-2 4th generation antibody/antigen screen blood (–); *Toxoplasma gondii* IgM, IgG (–)<br><br>Serum test: *Cryptococcal* antigen (–)
Hospital day 9<br>PCR stool: Enterovirus (–)	Hospital day 7<br>CSF tests at CDC: EEEV IgM MIA (+), PRNT 32 (+); *Baylisascaris procyonis* Ab immunoblot (–)<br><br>Serum tests at CDC: *Baylisascaris procyonis* Ab immunoblot (–)		Hospital day 8<br>Blood test: TB† (–)
Hospital day 11<br>Blood culture (–)	Hospital day 9<br>CSF bacterial culture (–)<br><br>CSF tests at Mayo Clinic: encephalopathy autoimmune evaluation (–)		Hospital day 9<br>CSF tests: fungal culture (–); *Cryptococcal* antigen (–); bacterial culture (–); flow cytometry (–); WNV IgG and IgM (–); and PCR for *Toxoplasma gondii* (–); enterovirus (–); CMV (–); VZV (–); and HSV (–).

Patient 1	Patient 2	Patient 3	Patient 4
			CSF IFA at an outside reference laboratory for EEEV IgG <1:4 (–), IgM <1:4 (–)
Hospital day 12<br>Lower respiratory culture: *Aspergillus* species (not fumigatus/flavus)<br><br>CSF tests: bacterial culture (–); Blood at UCSF: – metagenomics	Hospital day 10<br>Autopsy of the brain		Hospital day 10<br>Blood tests: culture ×2 (–)<br><br>CSF test: *Listeria* antibody (–)
			Hospital day 11<br>Serum test: *C. difficile* assay (–)<br><br>Blood test: TB† (–)<br><br>Sputum culture normal flora
			Hospital day 16<br>Deep wound cultures: fungal (–); AFB (–)
			Hospital day 21<br>*C. difficile* assay (–)<br><br>Blood tests: *Cryptococcal* antigen (–); *Coccidioides* antibody (–)<br><br>CSF test at Mayo Clinic: encephalopathy autoimmune evaluation (–)<br><br>CSF tests: Powassan virus IgM (–); *Cryptococcal* antigen (–); bacterial culture (–); fungal culture (–)
			Hospital day 23<br>Blood test: WNV antibody IgG and IgM (–)
			Hospital day 27<br>Sputum culture: normal flora
			Hospital day 31<br>Blood test: *Coccidioides* antibody (–)<br><br>Urine test: histoplasma antigen (–)<br><br>Serum test: *Cryptococcal* antigen (–)
			Hospital day 33<br>Blood culture 3/4 bottles Coagulase-negative *Staphylococcus*
			Hospital day 34<br>Blood culture 4/4 bottles Coagulase-negative *Staphylococcus*
			Hospital day 25<br>Blood culture (–)
			Hospital day 36<br>Blood culture 4/4 bottles *Candida albicans* (+)
			Hospital day 38<br>Stool test: *C. difficile* assay (–)
			Hospital day 39<br>Blood culture (–)
			Hospital day 40

Patient 1	Patient 2	Patient 3	Patient 4
			Blood culture (–)
			Hospital day 41<br>Blood tests: culture (–); ANCA (–); ANA (–); anti-RNP (–); anti-dsDNA (–); anti-Smith (–); SSA, SSB antigens (–)
			Hospital day 42<br>Blood culture (–)
			Hospital day 52<br>Blood culture (–)
			Hospital day 53<br>Peritoneal fluid: bacterial culture (–); fungal culture (–)<br><br>Sputum culture: *Pseudomonas aeruginosa* (+), *Escherichia coli* (+)

*Laboratory tests and date are reported. AFB, acid-fast bacilli; ANA, antinuclear antibody; ANCA, antineutrophil cytoplasmic antibody; CSF, cerebrospinal fluid; CMV, cytomegalovirus; EBNA-1, Epstein–Barr nuclear antigen 1; EBV, Epstein-Barr virus; EEEV, Eastern equine encephalitis virus; HSV, herpes simplex virus; IFA, indirect immunofluorescence assay; LCMV, lymphocytic choriomeningitis virus; MIA, microparticle immunoassay; NA, not applicable; PRNT, plaque reduction neutralization; TB, tuberculosis; UCSF, University of California, San Francisco; VCA, viral capsid antigen; VZV, Varicella-Zoster virus; –, negative; +, positive.
†QuantiFERON-TB GOLD (Qiagen, https://www.qiagen.com)

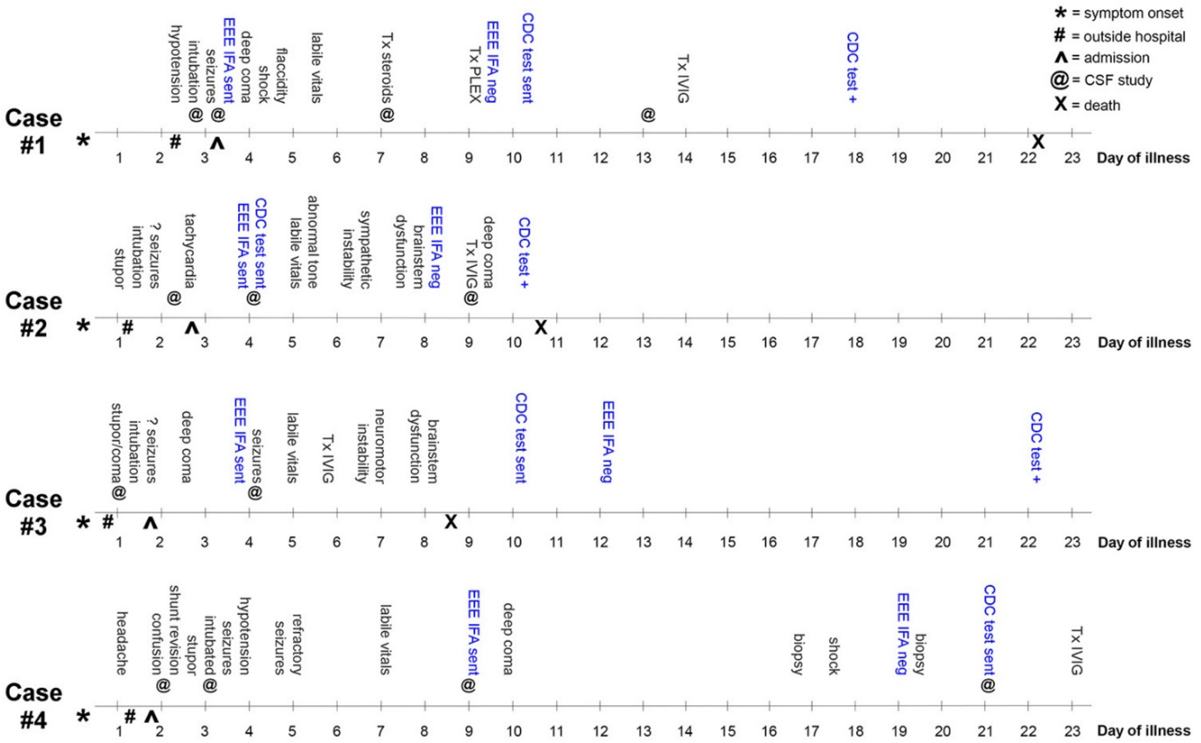

Appendix Figure 1. Progression, diagnosis, and clinical management in 4 human cases of Eastern equine encephalitis (EEE), Connecticut, USA, 2019. Clinical timelines for 4 EEE cases illustrate rapid worsening, empiric treatments and varying diagnostic results.

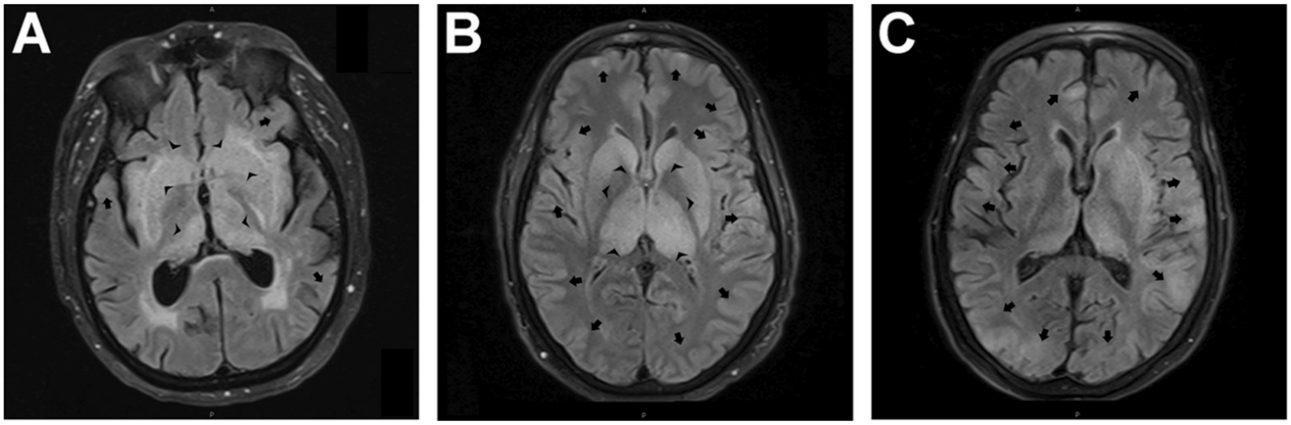

Appendix Figure 2. Magnetic resonance imaging (MRI) characterization of patterns of brain injury in 2 case-patients with Eastern equine encephalitis (EEE), Connecticut, USA, 2019. A) MRI axial section of case 1 on day 2 of hospitalization shows edema within and around the basal ganglia, thalamus, and basal forebrain, where EEE virus is known to invade early in the course of the disease. B) MRI axial section of case 1 on day 5 shows symmetric diffuse injury within the boundaries of cortex, striatum, thalamus, and regions of high metabolic demand. C) MRI axial section of case 4 on day 12 shows a pattern of diffuse, patchy widespread edema consistent with secondary inflammatory activation later during disease.

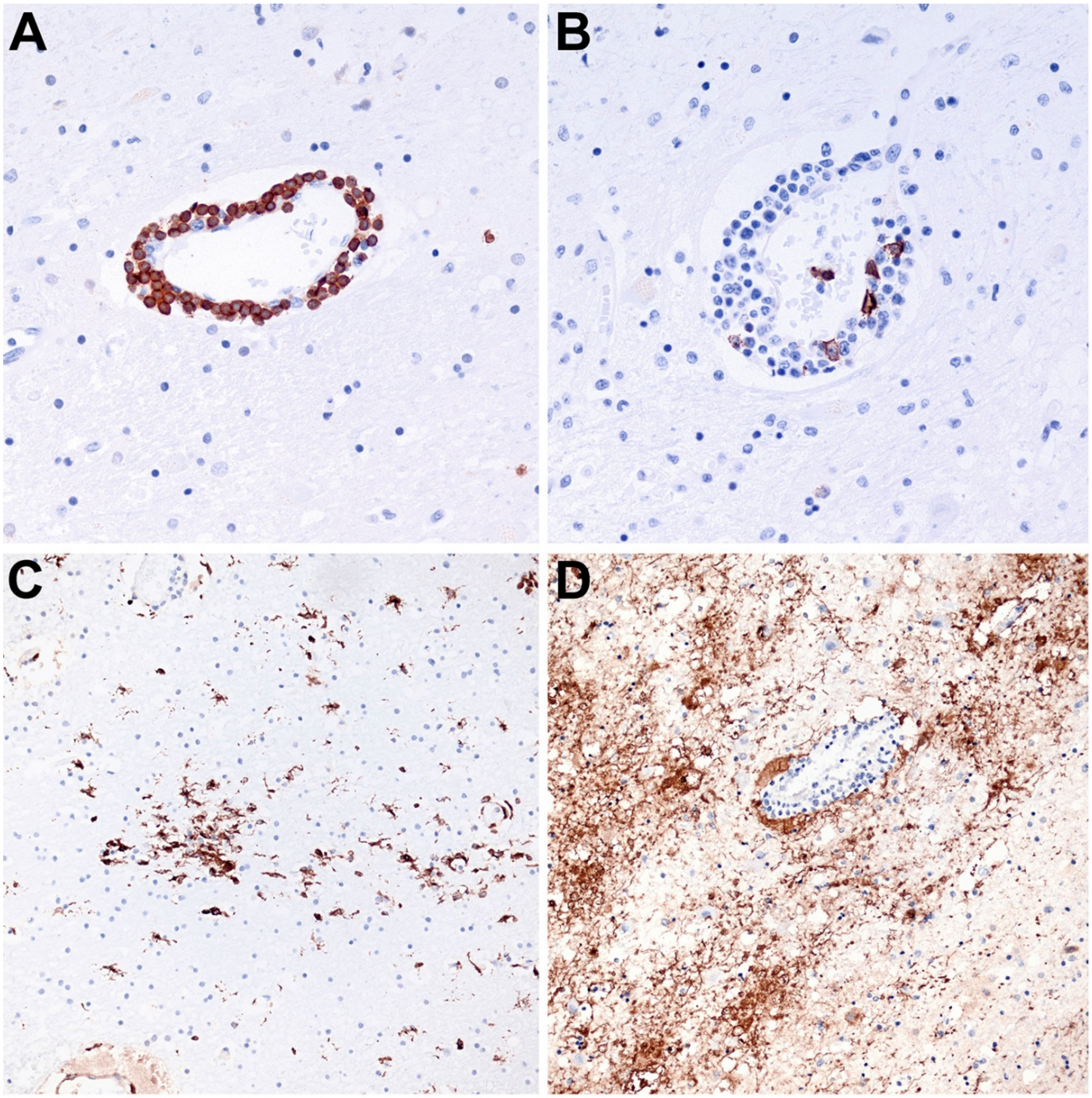

Appendix Figure 3. Representative histological characterization of Eastern equine encephalitis (EEE) inflammatory pathology in brain tissues, Connecticut, USA, 2019. A) CD3-stained photomicrograph shows perivascular lymphocytic cuffing from infiltration of T cells. Magnification ×400. B) CD20 staining demonstrates B cell infiltration ×400. C) CD163-stained photomicrograph shows nodular aggregates of microglia. Magnification ×40. D) GFAP staining demonstrates reactive astrocytes with patchy loss of neurons, signifying inflammatory activation in ischemic brain regions. Magnification ×40.

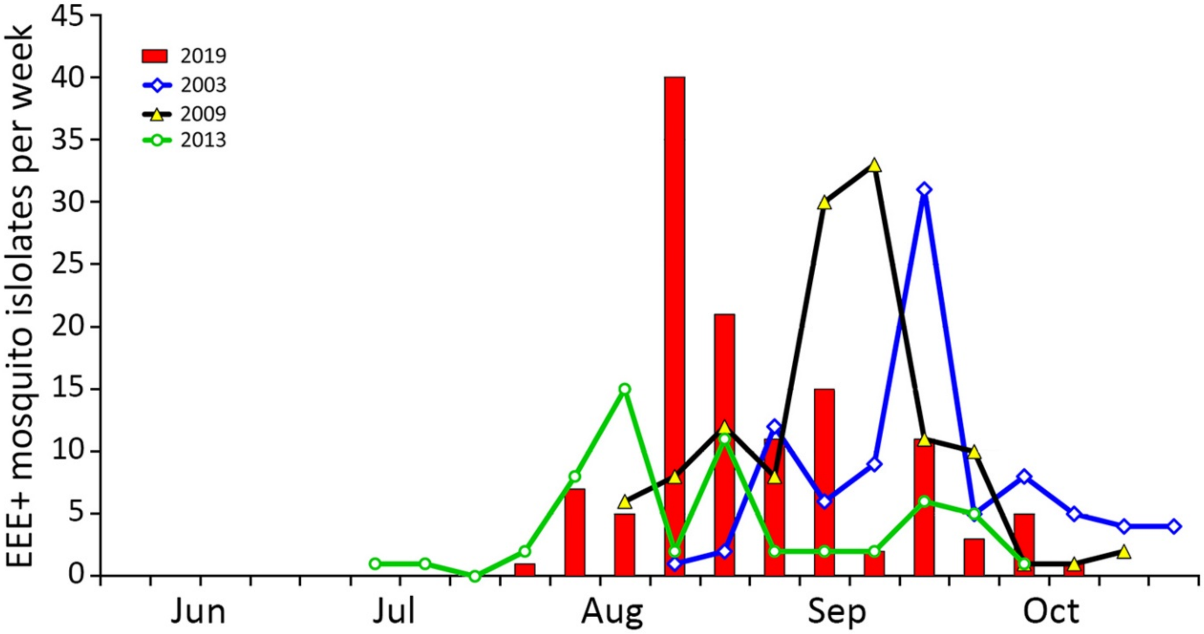

Appendix Figure 4. Weekly collection of mosquitoes that tested positive for Eastern equine encephalitis virus (EEEV+) during 4 years with increased EEEV activity, Connecticut, USA.

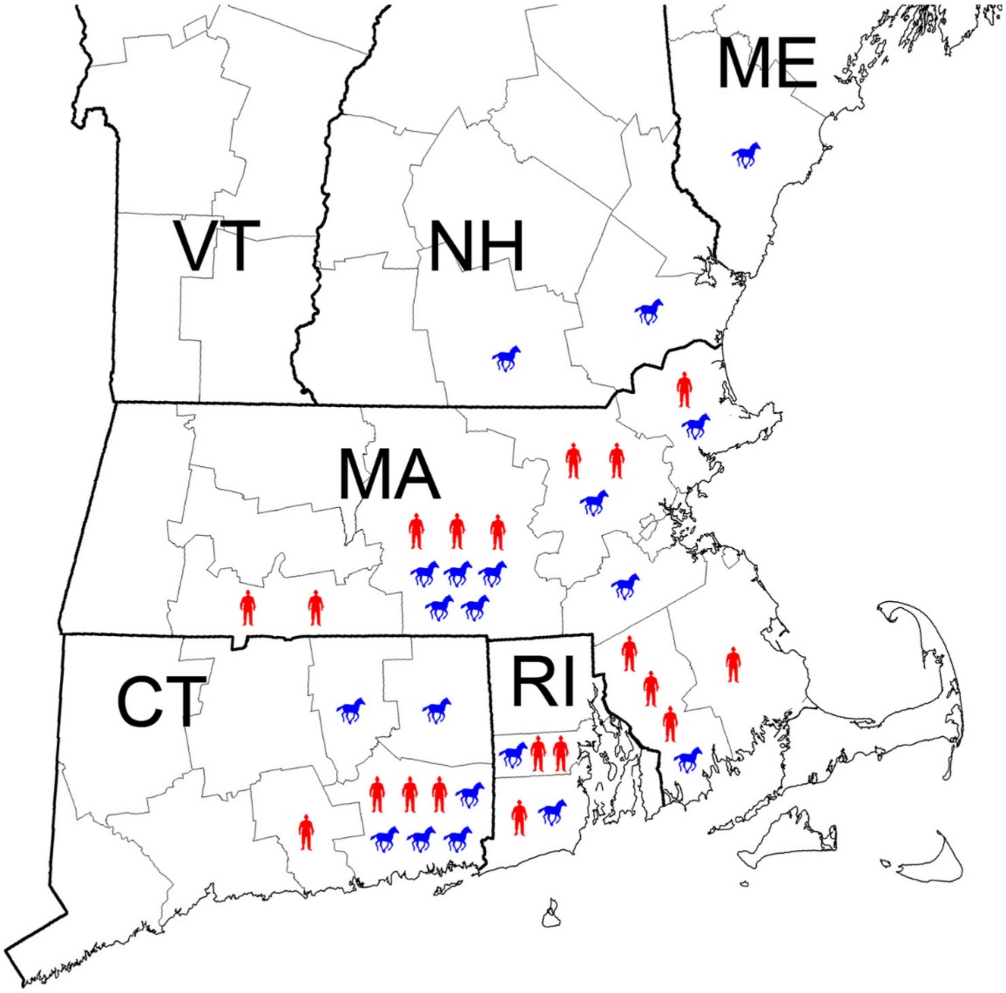

Appendix Figure 5. Geographic distribution of human and horse Eastern equine encephalitis cases in northeastern United States, 2019. CT, Connecticut; MA, Massachusetts; ME, Maine; NH, New Hampshire; RI, Rhode Island; VT, Vermont.
